# Unexpected phase transition sequence in the ferroelectric Bi_4_Ti_3_O_12_

**DOI:** 10.1107/S2052252519003804

**Published:** 2019-04-09

**Authors:** Yuan-Yuan Guo, Alexandra S. Gibbs, J. Manuel Perez-Mato, Philip Lightfoot

**Affiliations:** a School of Chemistry and EaStCHEM, University of St Andrews, St Andrews KY16 9ST, Scotland; b ISIS Facility, Rutherford Appleton Laboratory, Harwell Campus, Harwell OX11 0QX, UK; c Dept. of Condensed Matter Physics, University of the Basque Country UPV/EHU, Apartado 644, 48080 Bilbao, Spain

**Keywords:** perovskites, ferroelectrics, powder neutron diffraction

## Abstract

The high-temperature phase behaviour of the ferroelectric layered perovskite Bi_4_Ti_3_O_12_ has been re-examined by high-resolution powder neutron diffraction. Previous studies, both experimental and theoretical, had suggested conflicting structural models and phase transition sequences, exacerbated by the complex interplay of several competing structural instabilities. This study confirms that Bi_4_Ti_3_O_12_ undergoes two separate structural transitions from the aristotype tetragonal phase (space group *I*4*/mmm*) to the ambient-temperature ferroelectric phase (confirmed as monoclinic, *B*1*a*1). An unusual, and previously unconsidered, intermediate paraelectric phase is suggested to exist above *T*_C_ with tetragonal symmetry, space group *P*4*/mbm*. This phase is peculiar in displaying a unique type of octahedral tilting, in which the triple perovskite blocks of the layered structure alternate between tilted and untilted. This is rationalized in terms of the bonding requirements of the Bi^3+^ cations within the perovskite blocks.

## Abstract 2

A detailed powder neutron diffraction study reveals that the layered perovskite ferroelectric Bi_4_Ti_3_O_12_ undergoes an unusual phase transition sequence, exhibiting an unexpected and unique octahedral-tilted phase above *T*_C_.

## 1. Introduction   

**Figure 1:**
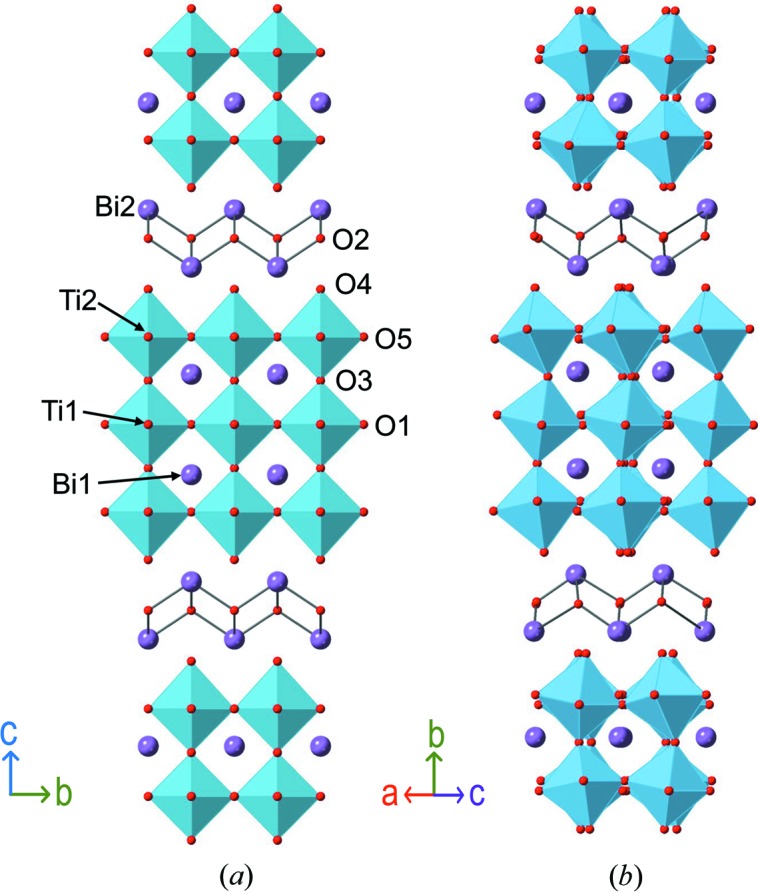
Crystal structures of (*a*) the aristotype (tetragonal, *I*4*/mmm*) and (*b*) the ambient-temperature (monoclinic, *B*1*a*1) phases.

Bi_4_Ti_3_O_12_ is an *n* = 3 member of the Aurivillius family of layered perovskite ferroelectrics (Aurivillius, 1950 ▸; Subbarao, 1962 ▸), (Bi_2_O_2_)*A*_*n*−1_ *B*_*n*_O_3*n*+1_. The symmetry of the ideal, paraelectric aristotype phase is tetragonal, space group *I*4/*mmm* (Fig. 1 ▸). A complex combination of octahedral tilting and atomic displacements leads to the room-temperature ferroelectric phase adopting the monoclinic space group *B*1*a*1 (standard setting *Pc*) (Rae *et al.*, 1990 ▸). Early variable-temperature crystallographic studies of Bi_4_Ti_3_O_12_ using electron microscopy (Nistor *et al.*, 1996 ▸), powder X-ray diffraction (PXRD) (Hirata & Yokokawa, 1997 ▸) and high-resolution powder neutron diffraction (PND) (Hervoches & Lightfoot, 1999 ▸) concluded that Bi_4_Ti_3_O_12_ did indeed adopt the aristotype *I*4/*mmm* structure at temperatures well above the ferroelectric Curie temperature, *T*_C_ ≃ 675°C. However, these analyses did not cover the nature of the evolution of the ambient-temperature monoclinic phase towards this aristotype tetragonal phase; for example, the present author’s previous study (Hervoches & Lightfoot, 1999 ▸) only reported data at two intermediate temperatures (500 and 650°C). Since that study, several authors have re-examined the nature of this complex structural evolution and differing structural models for various intermediate phases have been proposed. Kennedy and co-workers (Zhou *et al.*, 2003 ▸), using synchrotron PXRD, suggested an intermediate orthorhombic phase, space group *Cmce*, over a narrow temperature range above *T*_C_. In contrast, the optical microscopy measurements of Iwata *et al.* (2013 ▸) suggested a tetragonal phase above *T*_C_, but one of lower symmetry than the aristotype (either *P*4/*nnc* or *P*4_2_/*nmc*). We shall return to these studies later. Meanwhile, insights from theory, facilitated specifically by the combination of improvements to first-principles calculations and by the development of techniques for symmetry mode analysis, have helped to guide experimentalists towards possible crystallographic models that may not have been previously considered. Thus, Perez-Mato *et al.* (2008 ▸) illustrated the true complexity of the symmetry lowering from *I*4/*mmm* to *B*1*a*1 as consisting of the interplay of six distinct normal modes, three of which are necessary for the observed symmetry breaking. The presence of more than one primary symmetry-breaking mode at a single transition event can, in general, be considered as an exception to the Landau theory, and in this particular case the DFT calculations (Perez-Mato *et al.*, 2008 ▸), showed that the couplings among the relevant modes did not favour the simultaneous condensation of three primary order parameters. This gives rise to a scenario with several different possible pathways of symmetry descent from the aristotype phase to the experimentally observed ferroelectric phase at ambient temperature.

It is clear that considerable ambiguities still remain over the exact details of the thermal evolution of the crystallographic nature of Bi_4_Ti_3_O_12_. In order to shed further light on this, we have now carried out a more detailed high-resolution PND study, which supersedes our earlier work and provides significant new insights into this unusual phase transition behaviour. In particular, we make use of the methods of symmetry mode analysis (Perez-Mato *et al.*, 2010 ▸; Campbell *et al.*, 2006 ▸) to explore systematically the possible phase transition pathways on cooling from the aristotype *I*4/*mmm* phase. This study leads to a rather unexpected result.

## 2. Experimental   

### 2.1. Synthesis   

Bulk polycrystalline Bi_4_Ti_3_O_12_ was synthesized using a conventional mixed-oxide solid-state route. Stoichiometric amounts of Bi_2_O_3_ and TiO_2_ (99.9%, Alfa Aesar) were dried at 100°C for 48 h, ground and pressed into pellets of 13 mm diameter and approximately 2 mm thickness. Samples were subsequently heated at 700°C for 24 h, followed by 850°C for 24 h and cooled at a rate of 10°C min^−1^. Pellets were then re-ground to produce final powders suitable for characterization. Phase purity was confirmed by PXRD using Cu *K*α_1_ radiation with a wavelength of 1.5406 Å on a Panalytical EMPYREAN diffractometer (2θ step size of 0.017°, 1 h total scan time).

### 2.2. Powder neutron diffraction   

Time-of-flight neutron powder diffraction experiments were conducted using the high-resolution powder diffractometer at the ISIS neutron spallation source at the Rutherford–Appleton Laboratories. The polycrystalline sample (∼3 g) was sealed in a thin-walled quartz tube mounted in a cylindrical vanadium can. Data were collected at a series of temperatures commencing at 20°C, and subsequently at selected intervals for the temperature regime 150 ≤ *T* ≤ 1000°C. Each scan was counted for a 15 µAh incident proton beam (*ca* 20 min), except for longer scans (∼3 h, 120 µAh) at temperatures of 450, 615, 635, 655, 670, 685, 705, 850 and 1000°C.

### 2.3. Diffraction data analysis   

All PND data were analysed by Rietveld refinement using the *GSAS* software package with the *EXPGUI* interface (Toby, 2001 ▸). Refinement strategies were kept as uniform as possible across all datasets. For each dataset, two diffraction histograms were used [detector banks centred at 2θ = 168° (bank 1) and 90° (bank 2)]. The same set of profile parameters were refined in each case: twelve background coefficients and three peak-shape parameters for each histogram, three diffractometer constants and two histogram scale factors in total. In addition to these common parameters, appropriate lattice parameters, atomic positional coordinates and all isotropic atomic displacement parameters (ADPs) were refined; specific details are outlined for each crystallographic model in the relevant sections, as necessary. Symmetry mode analysis was carried out using the *ISODISTORT* suite (Campbell *et al.*, 2006 ▸). Further details of each of the crystallographic models, together with example Rietveld plots, are provided in the supporting information.

## 3. Results and discussion   

### 3.1. Ambient-temperature phase   

The aristotype structure (Fig. 1 ▸), space group *I*4/*mmm*, has the approximate unit cell metrics *a*_T_ ≃ 3.9 Å, *c*_T_ ≃ 33 Å. Both experimental (single-crystal X-ray, Rae *et al.*, 1990 ▸) and theoretical (Perez-Mato *et al.*, 2008 ▸) work have identified the monoclinic *B*1*a*1 model (Fig. 1 ▸) as the stable ground state of the ferroelectric phase (note: *B*1*a*1 is a non-standard setting of space group *Pc*, No. 7). This model has a unit cell of approximately double the volume of the aristotype (*a*_M_ ≃ *b*_M_ ≃ 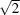 *a*_T_; *c*_M_ ≃ *c*_T_; β ≃ 90.0°). In PL’s earlier work (Hervoches & Lightfoot, 1999 ▸) this was approximated to an orthorhombic model, space group *B*2*eb*, with *a*_O_ ≃ *b*_O_ ≃ 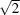 *a*_T_; *c*_O_ ≃ *c*_T_. The key difference between the (correct) monoclinic and (approximate) orthorhombic models lies in the presence of an additional ‘octahedral tilt’ mode in the former. Octahedral tilt modes are prevalent in the structural chemistry of perovskites, and it has been recognized that simultaneous condensation of two or more tilt modes can give rise to spontaneous polarization in layered perovskites, hence precise identification of these modes is critical to the understanding of ferroelectricity in layered perovskites (Benedek *et al.*, 2015 ▸). Although the additional tilt mode was not incorporated into the model used for the ambient-temperature phase in our earlier work (Hervoches & Lightfoot, 1999 ▸), with hindsight it can be seen that there is some evidence for its presence in the derived *B*2*eb* models presented in that work (*e.g.* anomalously large ADP of atom O1 in Tables 2 and 3). Hence, we first of all confirm that the *B*1*a*1 model is a more valid description of the ambient-temperature structure than the approximate *B*2*eb* model.

**Figure 2:**
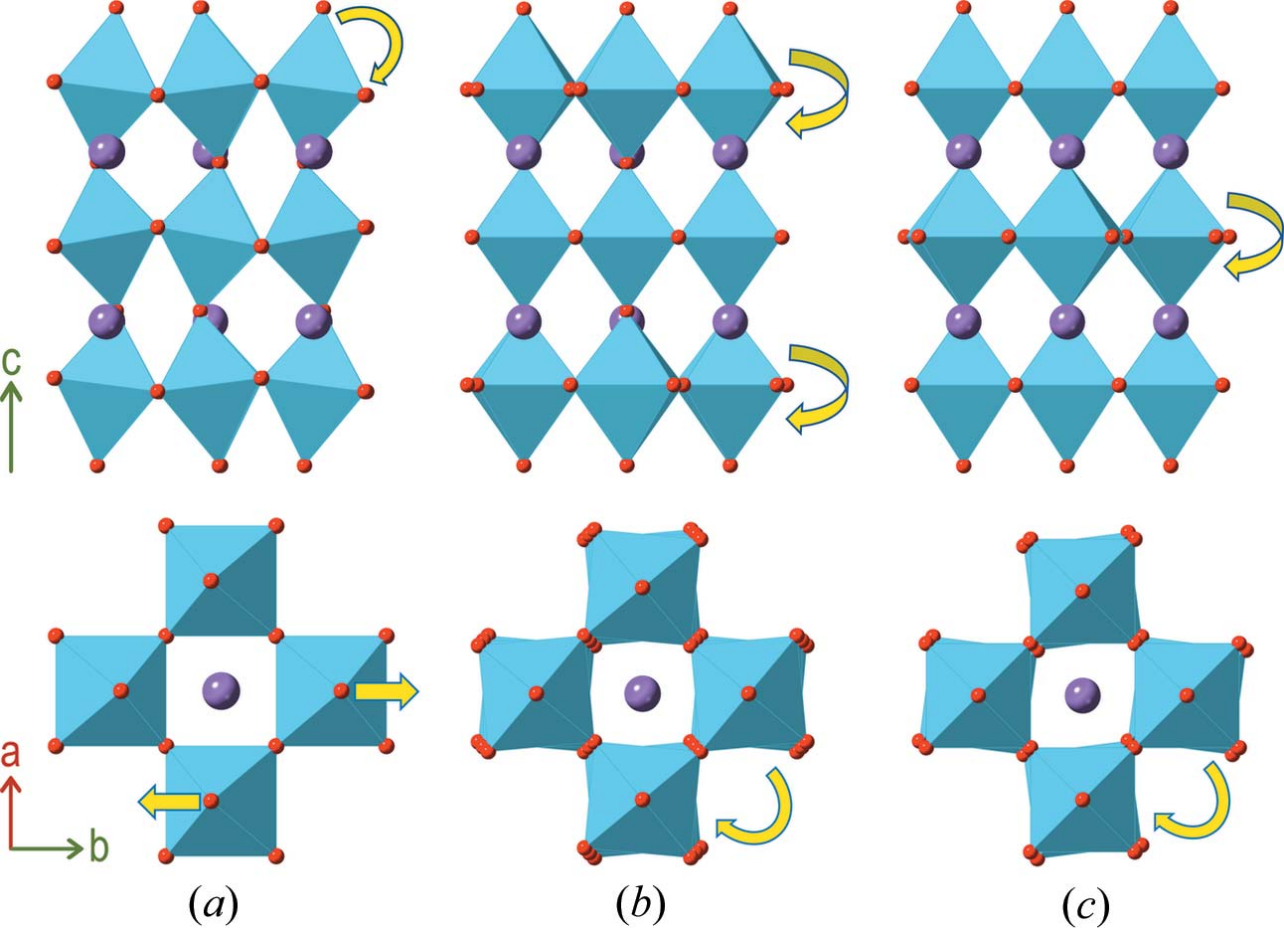
Details of the three individual tilt modes that contribute to the *B*1*a*1 model. Simulated mode amplitudes of 0.5 Å (*ISODISTORT*) have been used for illustration. Each plot shows an isolated triple-octahedral block view perpendicular and parallel to the unit cell axis *c* in the *B*1*a*1 setting. (*a*) The X_3_^+^ mode, showing out-of-plane (*ab*) tilting. (*b*) The X_1_^−^ mode, showing *anti*-phase rotation of the two outer octahedral layers only around *c*. (*c*) The X_2_^+^ mode, showing rotation of the inner octahedral layer only around *c*.

A starting model for the *B*1*a*1 phase was derived in two independent ways: the first was adapted from that reported by Rae *et al.* (1990 ▸) (we note that the model in ref. 3 uses an origin shift of Δ*y* = 0.25 relative to the setting used here). The second was derived from our own *B*2*eb* model (Hervoches & Lightfoot, 1999 ▸) by appropriate manual symmetry lowering. Careful Rietveld refinement of both models led to identical derived parameters and quality of fit, giving confidence in the reliability and robustness of this model. On close inspection of these models, using the *ISODISTORT* software, it was found that the most significant distortion modes, relative to the *I*4/*mmm* parent phase, were those that can be attributed to the *B*2*eb* distortion plus one additional mode (the previously mentioned additional octahedral tilt). There are many other distortion modes allowed in the *B*1*a*1 model, (the *B*1*a*1 model allows 55 variable atomic coordinates, whereas the *B*2*eb* model allows only 27), but since these were all of relatively minor magnitude, we have chosen to simplify this refinement model to allow easier comparison of the significant changes in the structure on proceeding to higher temperatures. Specifically, positional constraints were introduced such that all atoms conformed to the *B*2*eb* model, except O(1) and O(1)’, which are the ‘in-plane’ oxygen atoms of the middle layer of the perovskite blocks. This approximation does not significantly affect the derived parameters discussed below. Details of Rietveld refinement outcomes for all models discussed are given in Table 1 ▸. The most significant distortion modes, which consist of three different octahedral tilt modes, in addition to the polar atomic displacement mode, are shown in Fig. 2 ▸. In detail, there are three tilt modes, designated by the irreducible representation (*irrep*) notations X_1_^−^, X_3_^+^ and X_2_^+^. Further details of these modes are given in the supporting information. In addition to these three tilt modes, the dominant polar mode is designated Γ_5_^−^. This represents polarization along the *a* axis of the *B*1*a*1 setting of the unit cell. Although a polarization (Γ_3_^−^) is also allowed along the *c* axis, this was found to be relatively weak in preliminary refinements (most noticeably in minor polar displacements of the O atoms in the octahedra), and subsequently constrained to zero.

### 3.2. Thermal evolution of the structure; ambient temperature to *T*_C_   

**Figure 3:**
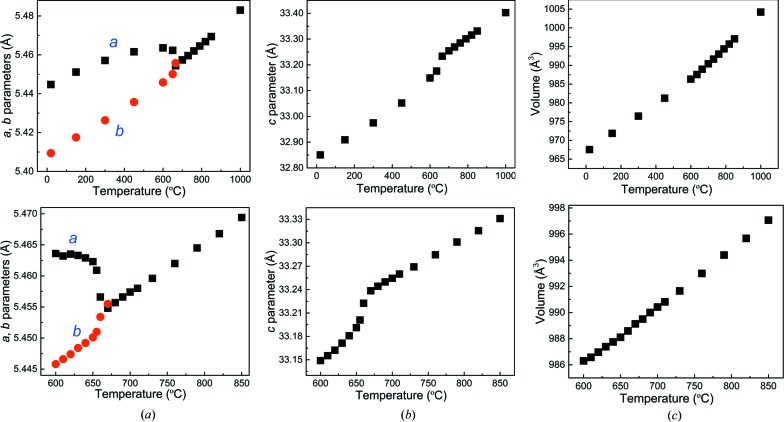
Full-range (top) and expanded (bottom) thermal evolution of the lattice metrics. (*a*) *a* and *b* parameters, (*b*) *c* parameter and (*c*) unit cell volume.

The partially constrained model described above for the ambient-temperature refinement was applied sequentially to all datasets proceeding upwards in temperature. Refinements proceeded smoothly and led to stable refinements, with comparable qualities of fit up to 670°C. Lattice metrics show a smooth convergence of the *a* and *b* unit cell parameters on increasing *T*, with an apparent coalescence at *T*_C_ (Fig. 3 ▸). Although the β angle remains very close to 90° throughout this temperature range (Fig. S1), and cannot be used in isolation as an unambiguous measure of crystal symmetry, the continued presence of key distortion modes (see below) confirms that monoclinic symmetry is retained all the way to *T*_C_.

**Figure 4:**
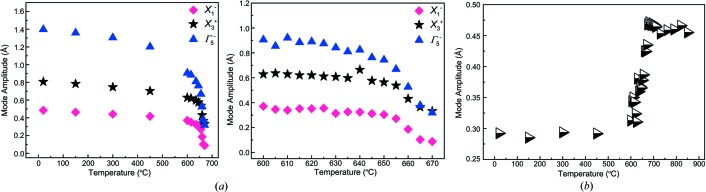
Thermal evolution of the four most significant modes: (*a*) X_1_^−^ and X_3_^+^ tilt modes and Γ_5_^−^ polar mode. Note that in the expanded plot on the right, all three modes feature a plateau towards *T*_C_. (*b*) X_2_^+^ tilt mode. The mode amplitudes are normalized relative to the common parent unit cell (parameter *A*_p_ in *ISODISTORT*).

*ISODISTORT* was used to derive mode amplitudes from each refinement: plots of the thermal evolution of the four most significant modes are given in Fig. 4 ▸. From these data, it can be immediately seen that the tilt modes X_1_^−^ and X_3_^+^, together with the polar mode Γ_5_^−^ show a gradual trend towards zero at *T*_C_ but, importantly, just below *T*_C_ a ‘plateauing’ is seen. In contrast, the X_2_^+^ mode shows a smaller variation throughout most of the temperature regime, followed by a more abrupt transition to a larger value at *T*_C_ and above.

The ‘plateauing’ behaviour of the X_1_^−^, X_3_^+^ and Γ_5_^−^ modes is suggestive of a first-order, rather than a continuous evolution of the structure through *T*_C_.

### 3.3. Nature of the phase immediately above *T*_C_   

**Figure 5:**
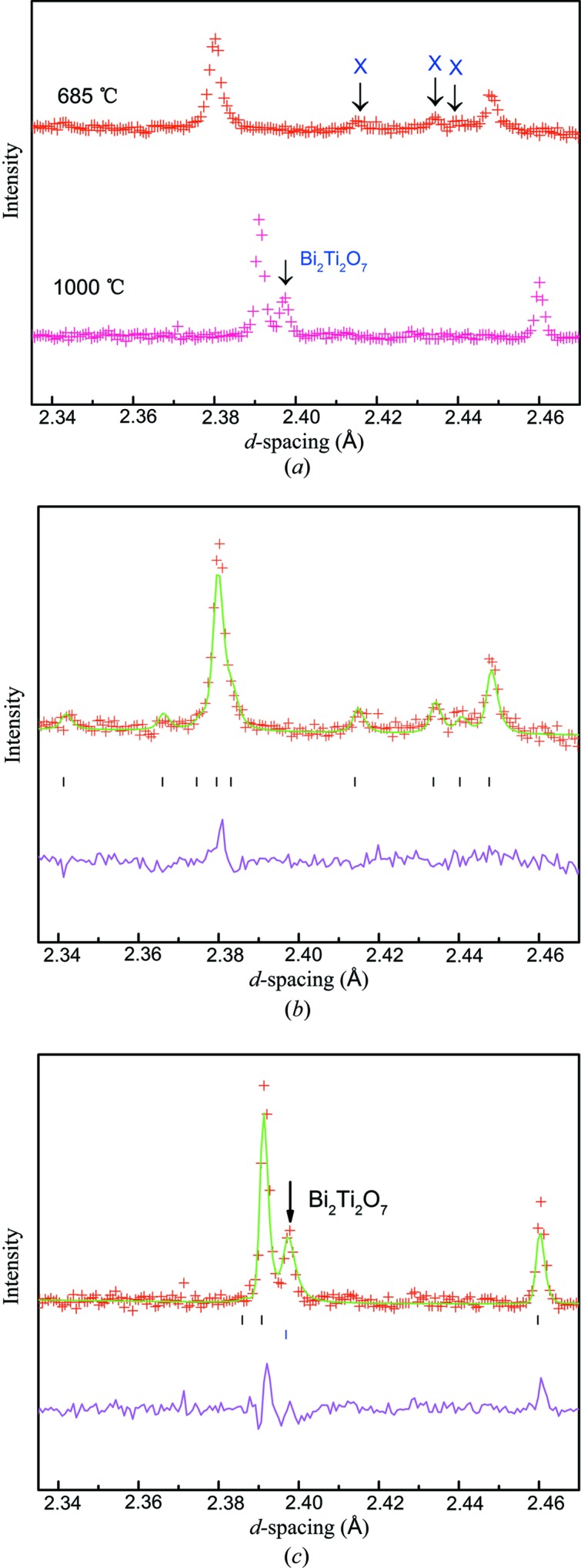
(*a*) Portion of the raw PND at 685°C (above), highlighting continued presence of the X-point peaks (arrowed) above *T*_C_, together with the corresponding region at 1000°C. (*b*) Corresponding Rietveld fit using the *P*4*/mbm* model. (*c*) Corresponding Rietveld fit at 1000°C (*I*4*/mmm* model): the peak at *d* ≃ 2.40 Å is a Bi_2_Ti_2_O_7_ impurity due to partial decomposition under these conditions.

Fig. 4 ▸ shows that the X_1_^−^, X_3_^+^ and Γ_5_^−^ modes display a decreasing and then ‘plateauing’ trend on heating towards *T*_C_, whereas the X_2_^+^ mode increases, and indeed becomes the strongest mode approaching *T*_C_. From this it might be inferred that, of the four largest ambient-temperature modes, the X_2_^+^ mode persists uniquely above *T*_C_. This is a curious and unexpected result, and a detailed comparison of selected models was undertaken to confirm it and establish a rationalization of the behaviour. Weak superlattice peaks at the X-point (1/2,1/2,0) of the parent *I*-centred tetragonal Brillouin zone can be clearly seen in the raw data (Fig. 5 ▸). The next step was to determine the possible space groups for the phase directly above *T*_C_, taking into account the continued presence of the X_2_^+^ mode, but with the absence of the X_1_^−^, X_3_^+^ and Γ_5_^−^ modes. Again, *ISODISTORT* was used to derive the simplest crystallographic models based on the requirement for the X_2_^+^ mode as the primary-order parameter. Three models are derived (see Table 1 ▸ and the supporting information for further details). Each of these retains a ‘doubled’ unit cell volume compared with the parent *I*4*/mmm* phase; *i.e.* 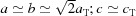 (unit cell axes for some models are switched in order to conform to standard space-group settings). As a comparison, models derived using *only* X_1_^−^ or X_3_^+^ modes were also refined for the 685°C dataset (Table 1 ▸). As a final check, two models were considered which permit X_2_^+^/X_1_^−^ or X_2_^+^/X_3_^+^ combinations. Neither of these produced significantly improved fits compared with the model chosen below (see supporting information).

These refinements firstly confirm the validity of the X_2_^+^ mode, (and not the X_1_^−^ or X_3_^+^ modes) in accounting for the presence of the weak X-point superlattice peaks above *T*_C_. Second, amongst the options displaying the X_2_^+^ mode, they clearly demonstrate a preference for the tetragonal (*P*4/*mbm*) model rather than the orthorhombic (*Cmce*) model; even the third *P*4/*mbm* model shown in Table 1 ▸, with constrained *U*_iso_ parameters, shows a significant improvement in fit due to the few additional structural variables.

**Figure 6:**
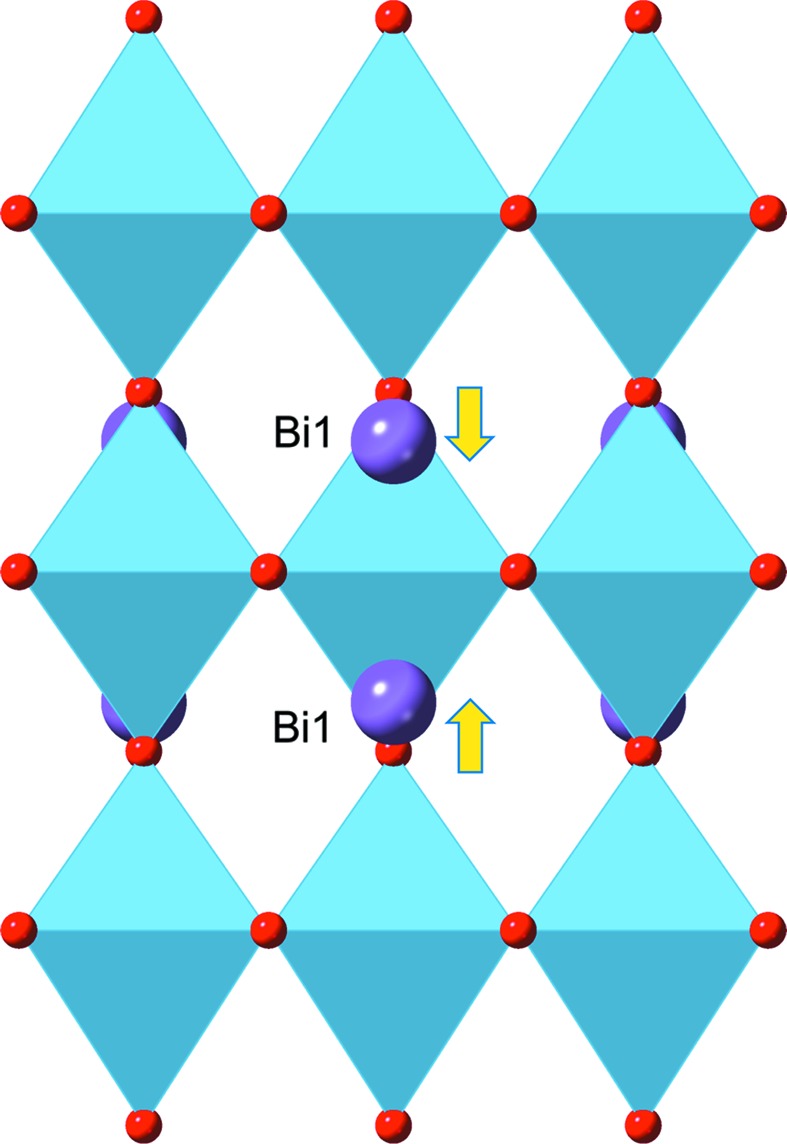
M_1_^+^ mode (present in the *P*4/*mbm* model but not in the *Cmce* model). The arrows describe a displacive degree of freedom of the Bi sites within the perovskite blocks: note that the Bi displacement alternates ‘towards’ or ‘away from’ the central octahedral site in alternate layers (see text).

The difference between the two models, *P*4/*mbm* and *Cmce*, is worthy of further description and analysis (see also supporting information). In both cases, the X_2_^+^ mode corresponds to a ‘rigid’ rotation of the central octahedral layer of the perovskite block around the ‘long’ unit cell axis (*i.e.* the *c* axis in the *B*1*a*1 and *P*4/*mbm* models). In the case of the *Cmce* model, this rotation occurs in *all* the perovskite blocks, acting in the same sense around *c* in each block. However, in the *P*4/*mbm* model the rotation occurs in only alternate perovskite blocks along *c*, with every other block having zero rotation. This mode therefore breaks the lattice centering, but maintains the tetragonal symmetry of the parent. This can be regarded as a ‘2**k**’ mode [**k1** = (1/2,1/2,0) and **k2** = (1/2,1/2,1)], whereas the mode of the same symmetry leading to the *Cmce* model has only a single **k** contribution (**k** = 1/2,1/2,1). Hence, although the crystal system may be regarded as ‘higher symmetry’ for the *P*4/*mbm* case, the *Cmce* model has the smaller (primitive) asymmetric unit cell and a smaller number of refineable atomic coordinates (Table 1 ▸). The ground-state model *B*1*a*1 resembles the *Cmce* model in this sense: *i.e.* its X_2_^+^ mode condenses at a single **k**-point. This means that there is no group–subgroup relationship between the *P*4/*mbm* and *B*1*a*1 models whereas there is such a relationship between the *Cmce* and *B*1*a*1 models. So, why do we propose that the *P*4/*mbm* model is preferred? First, there is no evidence from refined lattice parameter metrics for any deviation from tetragonal symmetry, although this in itself is insufficient evidence. More importantly, the tetragonal model has effectively ‘lower’ symmetry and allows additional distortion modes which are not permitted in the *Cmce* model. Specifically, there is a mode of M_1_^+^ symmetry which is active in the *P*4/*mbm* model. This mode affects primarily the Bi sites within the triple-perovskite blocks. As shown in Fig. 6 ▸, the M_1_^+^ mode allows displacement of these Bi atoms along the *c* axis, such that in blocks where the X_2_^+^ mode requires the octahedral rotation to be present, the Bi atoms are displaced *towards* the central octahedral layer, whereas in blocks with zero octahedral rotation, the Bi atoms are displaced *away from* the central layer. This subtle feature is presumably not coincidental, but allows the overall energetics of the system to be optimized by cooperation with the unusual tilt system. Further support for this correlation comes from bond-valence sum analysis, as described in the *Discussion* [Sec sec4].

The fact that this unusual type of transition (*i.e.* where a 2**k** mode leads to a situation of alternating ‘tilted’ and ‘untilted’ perovskite blocks) has apparently not been seen in other families of layered perovskites is perhaps due to the peculiar bonding preferences of the non-spherical Bi^3+^ cation; further work may be merited to explore this phenomenon. The final crystallographic model for the *P*4*/mbm* phase is presented in Table 2 ▸, and selected bond lengths in Table 3 ▸.

### 3.4. The parent phase   

The X-point reflections noted above are seen to persist to a temperature of at least 850°C, but disappear by 1000°C. We can therefore conclude that in the region 850–1000°C there is a final transition from *P*4*/mbm* to the parent structure *I*4/*mmm*. Only weak reflections violating this model (which are due to partial sample decomposition to the pyrochlore Bi_2_Ti_2_O_7_) are seen at 1000°C [Fig. 5 ▸(*c*)]. Unfortunately, we were not able to collect further datasets in this temperature region in order to define a more precise transition temperature, or to probe further the nature of this transition. The *I*4/*mmm* model refines straightforwardly and is in agreement with that previously presented in the work by Hervoches & Lightfoot (1999 ▸).

## 4. Discussion   

In our previous paper (Hervoches & Lightfoot, 1999 ▸) it was suggested that the driving force for any structural transition from the aristotype *I*4/*mmm* phase was most likely the result of significant underbonding at the A site of the perovskite block [Bi(1) site, Fig. 1 ▸]. Our current study supports that assertion, but leads to an unexpected means of relieving this underbonding: *via* activation of the 2**k** X_2_^+^ tilt mode (Fig. 4 ▸), together with the M_1_^+^ Bi displacement mode (Fig. 6 ▸). Bond-valence sum analysis (Brese & Keeffe, 1991 ▸) provides a semi-quantitative means of rationalizing this (we do not attempt a fully quantitative justification as the bond-valence method is semi-empirical and uses parameters derived from ambient-temperature crystal data). In Table 4 ▸, we compare bond lengths around the Bi(1) site for four *idealized* high-temperature models: (i) the parent *I*4*/mmm* phase; (ii) the *Cmce* phase (which allows a single **k** X_2_^+^ mode, but does not allow the M_1_^+^ Bi displacement mode); (iii) the *P*4*/mbm* phase, with only the 2**k** X_2_^+^ mode activated; and (iv) the *P*4*/mbm* phase, with both the 2**k** X_2_^+^ mode and the M_1_^+^ mode activated. Each of the lower-symmetry models are derived from the same *I*4*/mmm* parent, with fixed mode amplitudes of −0.5 Å for X_2_^+^ and 0.5 Å for M_1_^+^, derived from *ISODISTORT*.

What can be seen here is that the Bi(1) site is dramatically underbonded (bond valence sum, Σ*v* = 2.21 valence units) in the parent phase (the bond valence sum simply represents the nominal oxidation state of the bonded atom in this model, hence a Σ*v* of 3.0 would be expected for optimally bonded Bi^3+^). On permitting a small distortion due to the X_2_^+^ tilt mode within the *Cmce* model, it can be seen that eight of the twelve Bi—O bonds remain unaffected [those to O(3) and O(5); see Fig. 1 ▸], whereas the set of four bonds (at 2.96 Å) to the O(1) atom in the central octahedral layer split pairwise, ‘2 long + 2 short’. The net bonding environment around Bi(1) is improved by the introduction of the two shorter bonds (at 2.81 Å), which outweighs the weaker bonding to the two longer bonds (3.13 Å), resulting in a slight increase in the Σ*v* (2.23 v.u.). Incorporating the X_2_^+^ mode only into the *P*4*/mbm* model does not significantly change this (Σ*v* = 2.23 v.u.), but allowing the additional degree of freedom from inclusion of the M_1_^+^ mode makes a significant difference, the Bi(1) site within the ‘tilted’ layer benefiting from an increase in Σ*v* to 2.45 v.u. [and with only a minor disadvantage in bonding to the other Bi(1) site].

The above argument is only intended to illustrate the general features of the differing distortion modes and their effect on the bonding around the Bi(1) site. The observed magnitudes of the X_2_^+^ and M_1_^+^ modes in our final refinement of the *P*4/*mbm* model (Tables 2 and 3) are of the order of −1.0 and 0.3 Å, respectively, leading to actual Σ*v* values of 2.38 and 2.39 v.u. In comparison, our experimentally determined Σ*v* for the Bi(1) site in the *Cmce* model is only 2.33, confirming the validity of the above generic argument.

The present study demonstrates that Bi_4_Ti_3_O_12_ undergoes a highly unusual structural evolution *versus* temperature, displaying two distinct phase transitions, incorporating several competing active modes. Previous experimental studies have failed to recognize the details of these modes, and hence have proposed incorrect or incomplete models for the intermediate phase (the *P*4*/mbm* phase in this study). Our own earlier study (Hervoches & Lightfoot, 1999 ▸) did not see the intermediate phase, in part due to the larger temperature intervals used in that experiment. Two more recent studies are also worthy of comment. Kennedy and co-workers (Zhou *et al.*, 2003 ▸) proposed, from synchrotron PXRD, an intermediate orthorhombic model just above *T*_C_ (in the temperature interval 675 < *T* < 695°C). It is significant to note, however, that despite the same apparent space group (*Cmce*), this is *not* the same model as that considered in the present work. Kennedy’s suggestion (a full model was not proposed) arises from the *I*4*/mmm* parent *via* condensation of the X_3_^+^ mode, not the X_2_^+^ mode, and was based on an implicit assumption of a single **k** mode. Hence, the unit cell requires a different setting of the space group *Cmce* to the one considered here: the two alternatives can be seen in the group–subgroup graph suggested in Perez-Mato *et al.* (2008 ▸) and are also shown in Table S1 of the supporting information. Iwata’s study, using optical microscopy on single crystalline samples, identified the tetragonal (not orthorhombic) nature of the intermediate phase at 800°C, and indeed this offers support to our own proposed model having tetragonal symmetry. However, they failed to identify the superlattice due to the X-point distortion. Instead, that study proposed an M-point distortion from the *I*4*/mmm* parent, leading to a unit cell of the same size as the parent (*a*_T_ ≃ 3.9 Å, *c*_T_ ≃ 33 Å), but with symmetry lowered from body-centred to primitive. Unfortunately, a full structural model was again not proposed, nor details of the specific mode(s) underlying the proposed distortion. However, it is clear that in a model with only Γ and M-points active, there can be no octahedral tilting, only atomic displacements along the *c* axis.

It is perhaps not surprising, with hindsight, that the subtleties of the *P*4*/mbm* option were missed in all of the previous studies; its identification relies on careful observation and modelling of the weak X-point peaks above *T*_C_, for which neutron diffraction is so well suited.

Two structural transitions are observed in Bi_4_Ti_3_O_12_ in this study; *I*4*/mmm* to *P*4*/mbm* in the region 850–1000°C and *P*4*/mbm* to *B*1*a*1 at *T*_C_ ≃ 675°C. In the case of the *I*4*/mmm* to *P*4*/mbm* transition, the scenario is the usual Landau-type transition with a single *irrep* for the order parameter or distortion, which is responsible for the symmetry break. This primary mode corresponds to the X_2_^+^ *irrep* and involves its two distinct wavevectors. But, as an induced effect, a secondary mode with symmetry M_1_^+^ is also present. The X_2_^+^ primary distortion mode is essentially the same as that present in the lower-temperature phase (reducing its symmetry from orthorhombic to monoclinic), but with the difference that in this intermediate phase the modes corresponding to the two wavevectors, **k1** and **k2**, of the *irrep* are concomitantly condensed, and thus maintain a tetragonal symmetry, whereas in the monoclinic phase at lower temperatures only one of the modes associated with one of the two wavevectors is involved. Only the simultaneous condensation of the two waves allows the presence of the mentioned secondary M_1_^+^ mode, and this could be the fundamental reason favouring this phase. Phenomenologically, the condensation of the M_1_^+^ distortion can be regarded as a consequence of a coupling in a *trilinear* fashion (Benedek *et al.*, 2015 ▸; Extebarria *et al.*, 2010 ▸), *i.e.* the free energy is lowered by a term of the form 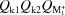, where *Q* represents the magnitude of each mode.

From theoretical DFT calculations Perez-Mato *et al.* (2008 ▸) concluded that a direct transition from the parent phase *I*4/*mmm* to the monoclinic phase with symmetry *B*1*a*1, as generally assumed, was very unlikely, as the energy landscape did not favour the necessary simultaneous condensation of the three primary distortion modes associated with different *irreps* that are present in the monoclinic phase. Such ‘avalanche’ first-order transitions are known for two order parameters, but not for three, and in the present case there is no indication in the mode couplings that would promote such an exceptional scenario. The existence of an intermediate phase where one of the order parameters previously condenses solves this puzzle, and the second transition between the *P*4*/mbm* and *B*1*a*1 symmetries is now reduced to the simultaneous activation of two additional primary distortion modes, together with a change of direction (change from a 2**k** to a 1**k** distortion) of the order parameter associated with the intermediate phase. This second phase transition is then analogous to the one in Aurivillius compounds with perovskite blocks with only two layers (Perez-Mato *et al.*, 2004 ▸), the difference being the additional previous condensation of the X_2_^+^ mode, which involves only the central octahedral layers and it is therefore specific to materials with three layers in the perovskite blocks. The instability of this additional distortion was shown (Perez-Mato *et al.*, 2008 ▸) to be very strong, comparable with the polar strongest one, and therefore its condensation at a higher temperature than the rest is not unreasonable, but the unexpected feature is the involvement of the two wavevector branches of this distortion, which was overlooked in previous theoretical calculations as a competitive configuration of the distortion.

From the above analysis the transition at *T*_C_ takes place between two phases that are not group–subgroup related, and therefore it is necessarily a first-order or discontinuous transition. This is supported by the ‘plateau’ behaviour of the tilts and polar mode just below *T*_C_ (Fig. 4 ▸). The condensation of the additional modes is concomitant with the disappearance of the M_1_^+^ mode and the reduction to zero of the amplitude for one of the two wavevectors involved in the X_2_^+^ distortion. The X_2_^+^ distortion remains in the ground state of the system, but with its amplitude significantly reduced due to its unfavourable coupling (Perez-Mato *et al.*, 2008 ▸), with the other spontaneous distortions. Even if the intermediate phase is the result of a 1**k** X_2_^+^ distortion, resulting in a *Cmce* symmetry (instead of the *P*4/*mbm* space group proposed here) then, although there would be a group–subgroup relation between the symmetries of the intermediate and the monoclinic phase, the transition would also be first order, since this symmetry break implies an avalanche transition, with two *irreps* being activated simultaneously. We note that there is no rule forbidding an intermediate phase with a less dense set of lattice translations (and a larger primitive unit cell) than the lower-temperature phase, and this always happens in cases where the high rotational symmetry is maintained by combining several distortion modes with symmetry-related **k**-vectors (multi-**k** case).

## 5. Conclusions   

In conclusion, we have shown that Bi_4_Ti_3_O_12_ undergoes a highly unusual phase evolution *versus* temperature. The aristotype tetragonal phase (space group *I*4/*mmm*) undergoes a two-step transition to the monoclinic ground state (space group *B*1*a*1) on cooling. At temperatures still well above *T*_C_ (∼675°C), an octahedral tilt mode described by *irrep* X_2_^+^ acts to reduce the symmetry to an intermediate centrosymmetric phase. The activation of this mode above *T*_C_ has not been considered in previous studies. Of the two simplest models considered, a tetragonal phase with symmetry *P*4/*mbm* is determined to be the most likely. Significantly, the X_2_^+^ mode present in this model affects octahedral tilts in alternating perovskite-like blocks along the *c* axis, a phenomenon which has not been seen in other families of layered perovskites as far as we are aware. This can be rationalized in terms of the most favourable way to optimize bonding interactions around the Bi atoms within the perovskite blocks. On lowering the temperature through *T*_C_, two further octahedral tilt modes are activated simultaneously, coupled with the polar displacive mode, leading directly to the monoclinic (*B*1*a*1) ground state. Despite the fact that three significant modes appear together below *T*_C_, the polar Γ_5_^−^ mode appears dominant, and Bi_4_Ti_3_O_12_ may hence be described as a proper ferroelectric.

The research data supporting this publication can be accessed at https://doi.org/10.17630/b7c11904-b4b0-4acf-a02a-b1e53aec3672.

## Supplementary Material

Crystal structure: contains datablock(s) BI4TI3O12-655_publ, BI4TI3O12-655_overall, BI4TI3O12-655_phase_1, BI4TI3O12-655_p_01, BI4TI3O12-655_p_02, BI4TI3O12-20_overall, BI4TI3O12-20_phase_1, BI4TI3O12-20_p_01, BI4TI3O12-20_p_02, BI4TI3O12-685-X2-CMC_overall, BI4TI3O12-685-X2-CMC_phase_1, BI4TI3O12-685-X2-CMC_p_01, BI4TI3O12-685-X2-CMC_p_02, 1000-TWOPHASE_overall, 1000-TWOPHASE_phase_1, 1000-TWOPHASE_phase_2, 1000-TWOPHASE_p_01, 1000-TWOPHASE_p_02, BI4TI3O12_P4MBM-3_overall, BI4TI3O12_P4MBM-3_phase_1, BI4TI3O12_P4MBM-3_p_01, BI4TI3O12_P4MBM-3_p_02, BI4TI3O12_P4NBM_overall, BI4TI3O12_P4NBM_phase_1, BI4TI3O12_P4NBM_p_01, BI4TI3O12_P4NBM_p_02, BI4TI3O12_P42NCM_overall, BI4TI3O12_P42NCM_phase_1, BI4TI3O12_P42NCM_p_01, BI4TI3O12_P42NCM_p_02. DOI: 10.1107/S2052252519003804/lc5103sup1.cif

CIF format file for idealised Cmce model, X2+ mode. DOI: 10.1107/S2052252519003804/lc5103sup2.txt

CIF format file for idealised P4/mbm model, X2+ mode). DOI: 10.1107/S2052252519003804/lc5103sup3.txt

CIF format file for idealised P4/mbm model, X2+, M1+ modes). DOI: 10.1107/S2052252519003804/lc5103sup4.txt

ISODISTORT output for B1a1 model (78 variables) at RT. DOI: 10.1107/S2052252519003804/lc5103sup5.txt

ISODISTORT output for B1a1 model (101 variables) at RT. DOI: 10.1107/S2052252519003804/lc5103sup6.txt

ISODISTORT output for P4/mbm model at 685C. DOI: 10.1107/S2052252519003804/lc5103sup7.txt

Supporting information file. DOI: 10.1107/S2052252519003804/lc5103sup8.pdf

Unexpected phase transition sequence in the ferroelectric Bi4Ti3O12 (dataset): https://doi.org/10.17630/b7c11904-b4b0-4acf-a02a-b1e53aec3672

CCDC references: 1907946, 1907947, 1907948, 1907949, 1907950, 1907951, 1907952, 1907953

## Figures and Tables

**Table 1:** Details of the crystallographic models and Rietveld refinements for each of the three observed phases *N*_xyz_ is the number of refined atomic coordinates (in the case of the *B*1*a*1 model, constraints have been added – see text. In all other cases, all atomic coordinates were refined freely). *N*_tot_ is the total number of refined parameters (see text).

Temp (°C)	Space group	Significant modes	Unit cell (Å, °)	*N*_*xyz*_	*N*_tot_	χ^2^/*R*_wp_
20	***B*1*a*1**	X_1_^−^, X_3_^+^, X_2_^+^, Γ_5_^−^	*a* = 5.4447 (1), *b* = 5.4094 (1), *c* = 32.8504 (7), β = 90.047 (3)	30	78	11.86,† 2.35
				55	101	9.83, 2.15
685	***P*4*/mbm***	X_2_^+^, M_1_^+^	*a* = 5.4563 (1), *c* = 33.2475 (4)	18	72	6.30, 2.39
				18	68	6.38,‡ 2.41
				18	64	6.59, 2.45
	*Cmce*	X_2_^+^	*a* = 33.2475 (5), *b* = 5.4561 (4), *c* = 5.4566 (4)	11	58	7.01, 2.53
	*P*4_2_/*ncm*	X_3_^+^	*a* = 5.4562 (1), *c* = 33.2479 (5)	15	65	8.50, 2.78
	*P*4*/nbm*	X_1_^−^	*a* = 5.4563 (1), *c* = 33.2476 (5)	16	70	7.95, 2.69
1000	***I*4*/mmm***	None	*a* = 3.8771 (1), *c* = 33.4026 (4)	6	52	8.50, 3.06

† The two refinements shown are those with ‘partially constrained’ and ‘fully independent’ coordinates, described in the text.

‡ In the case of the *P*4*/mbm* model, the refinement was carried out (i) with all *U*_iso_ values independent (χ^2^ = 6.30), (ii) with the cation *U*_iso_s treated as ‘paired’ according to parent atom type (χ^2^ = 6.38), (iii) with all the *U*_iso_s treated as ‘paired’ according to parent atom type (χ^2^ = 6.59); the second model is reported in Tables 2 and 3.

**Table 2:** Refined crystallographic model for the intermediate *P*4/*mbm* phase at 685°C, lattice parameters: *a* = 5.4563 (1), *c* = 33.2475 (4) Å

Atom	*x*	*y*	*z*	*U*_iso_ (Å^2^)
Bi1(1)†	0.0000	0.5000	0.5650 (1)	0.0452 (5)
Bi1(2)	0.0000	0.0000	0.0715 (1)	0.0452 (5)
Bi2(1)	0.0000	0.5000	0.7106 (2)	0.0356 (5)
Bi2(2)	0.0000	0.0000	0.2113 (2)	0.0356 (5)
Ti1(1)	0.0000	0.5000	0.0000	0.019 (1)
Ti1(2)	0.0000	0.0000	0.5000	0.019 (1)
Ti2(1)	0.0000	0.5000	0.8705 (3)	0.0161 (7)
Ti2(2)	0.0000	0.0000	0.3717 (3)	0.0161 (7)
O1(1)	0.7531 (9)	0.2531 (9)	0.0000	0.052 (2)
O1(2)	0.3135 (7)	0.8135 (7)	0.5000	0.023 (1)
O2(1)	0.7497 (5)	0.2497 (5)	0.2516 (2)	0.0271 (7)
O3(1)	0.0000	0.5000	−0.0592 (3)	0.073 (3)
O3(2)	0.0000	0.0000	0.4412 (3)	0.056 (2)
O4(1)	0.0000	0.5000	0.8175 (2)	0.052 (2)
O4(2)	0.0000	0.0000	0.3174 (2)	0.042 (2)
O5(1)	0.7473 (6)	0.2473 (6)	0.6164 (2)	0.052 (2)
O5(2)	0.2517 (6)	0.7517 (5)	0.8822 (1)	0.029 (1)

† Cation *U*_iso_ values were constrained ‘pairwise’ according to the parent phase symmetry.

**Table 3:** Key bond lengths for the intermediate *P*4/*mbm* phase at 685°C

Bond	Length (Å)	Bond	Length (Å)
Bi1(1)—O1(2) × 2	2.596 (5)	Ti1(1)—O1(1) × 2	1.905 (7)
Bi1(1)—O1(3) × 2	2.736 (1)	Ti1(1)—O1(1) × 2	1.953 (7)
Bi1(1)—O5(1) × 2	2.593 (6)	Ti1(1)—O3(1) × 2	1.969 (11)
Bi1(1)—O5(1) × 2	2.562 (6)	Ti1(2)—O1(2) × 4	1.990 (1)
Bi1(2)—O3(1) × 4	2.759 (2)	Ti1(2)—O3(2) × 2	1.955 (10)
Bi1(2)—O5(2) × 4	2.468 (4)	Ti2(1)—O3(1)	2.337 (14)
Bi2(1)—O2(1) × 2	2.305 (6)	Ti2(1)—O4(1)	1.761 (13)
Bi2(1)—O2(1) × 2	2.301 (6)	Ti2(1)—O5(2) × 2	1.980 (5)
Bi2(1)—O4(2) × 3	2.882 (3)	Ti2(1)—O5(2) × 2	1.955 (5)
Bi2(2)—O2(1) × 4	2.349 (5)	Ti2(2)—O3(2)	2.310 (13)
Bi2(2)—O4(1) × 4	2.891 (3)	Ti2(2)—O4(2)	1.805 (13)
		Ti2(2)—O5(1) × 4	1.969 (2)

**Table 4:** Comparison of the bonding environments of Bi(1) for selected idealized models For the *P*4*/mbm* models two different Bi(1) sites are generated in alternate perovskite blocks; these are shown in separate rows. Σ*v* is the bond valence sum for Bi(1), taking into account the 12 Bi—O bonds. For each, bond, the bond valence is *v* = exp((R_0_ − *d*)/*b*), where *d* is the individual bond length, *R*_0_ is a constant for a particular bond type (*e.g.* 2.09 Å for Bi—O) and *b* = 0.37 Å, a universal constant.

Bond (Å)	*I*4*/mmm* (i)	*Cmce* (ii)	*P*4*/mbm* (iii)	*P*4*/mbm* (iv)
Bi1—O5	2.55 × 4	2.55 × 4	2.55 × 4	2.67 × 4
			2.55 × 4	2.44 × 4
Bi1—O3	2.75 × 4	2.75 × 4	2.75 × 4	2.73 × 4
			2.75 × 4	2.77 × 4
Bi1—O1	2.96 × 4	2.81 × 2; 3.13 × 2	2.81 × 2; 3.13 × 2	2.67 × 2; 3.01 × 2
			2.96 × 4	3.10 × 4
Σ*v* (Bi1)	2.21	2.23	2.23 2.21	2.13 2.45
