# Supplementary material for: Unexpected phase transition sequence in the ferroelectric Bi4Ti3O12
Source: IUCrJ. 2019 Apr 9;6(Pt 3):438–46. doi: 10.1107/S2052252519003804 (PMC6503926; doi:10.1107/S2052252519003804)
Supplement: Supplementary file 8 [file m-06-00438-sup8.pdf]

IUCrJ

Volume 6 (2019)

Supporting information for article:

Unexpected Phase Transition Sequence in the Ferroelectric Bi4Ti3O12

Yuan-Yuan Guo, Alexandra S. Gibbs, J. Manuel Perez-Mato and Philip Lightfoot

Table S1 Crystallographic models derived from the *I*4/*mmm* parent structure by action of each significant tilt mode or selected combinations.

Table S2 Constraints applied to the ‘simplified’ *B*1*a*1 model discussed in the text, such that all atoms except O1/O1’ confirm to the ideal *B*2*eb* model.

Figure S1: Temperature dependence of β angle for the monoclinic phase below T_C.

Figure S2. Crystal structures of the *P*4/*mbm* and *Cmce* models for the intermediate phase at 685 °C, with atomic numbering for the *P*4/*mbm* phase, and highlighting the differing X_2^+ modes and the M_1^+ modes for the *P*4/*mbm* model.

Figure S3. Rietveld plots, *B*1*a*1 ‘constrained’ model at 20 °C

Figure S4. Rietveld plots, *B*1*a*1 ‘constrained’ model at 655 °C

Figure S5. Rietveld plots, *P*4/*mbm* model at 685 °C

Figure S6. Rietveld plots, *I*4/*mmm* model (+ $Bi_2Ti_2O_7$ impurity) at 1000 °C

Separate Files:

1. ISODISTORT output for refined *B*1*a*1 models (‘constrained’ and ‘independent’) at 20 °C (Isodistort_B1a1_RT_78v; Isodistort_B1a1_RT_101v) and refined *P*4/*mbm* model (68 variables) at 685 °C (Isodistort_P4mbm_685C_68v).
2. CIF files for *B*1*a*1 (constrained) model at 20 °C (B1a1_RT_78v) and 655 °C (B1a1_655C_78v).
3. CIF files for comparative refined models at 685 °C (Table 1): *P*4/*mbm*, 68 variables (P4mbm_685C), *Cmce* (Cmce_685C) $P4_2/ncm$ (P42ncm_685C) and *P*4/*nbm*, (P4nbm_685C).
4. CIF files for idealized models described in Table 4 (Cmce_X2+, P4mbm_X2+, P4mbm_X2+_M1+).
5. CIF file for *I*4/*mmm* model at 1000 °C, with impurity phase included (I4mmm_1000C).

Table S1 Crystallographic models derived from the *I4/mmm* parent structure by action of each significant tilt mode or selected combinations.

Mode	OPD	Space group	basis	origin	s,i	k-active
**X_3^+**	(a,a)	*$P4_2/ncm$*	(1,1,0) (-1,1,0) (0,0,1)	(0,0,0)	4,4	(1/2,1/2,0), (1/2,1/2,1)
**X_3^+**	(o,a)	*Cmce*	(1,1,0) (0,0,1) (1,-1,0)	(0,0,0)	2,4	(1/2,1/2,1)
**$^{\$}X_3^+$**	(a,b)	*Pccn*	(1,1,0) (-1,1,0) (0,0,1)	(0,0,0)	4,8	(1/2,1/2,0), (1/2,1/2,1)
**X_1^-**	(a,a)	*P4/nbm*	(1,1,0) (-1,1,0) (0,0,1)	(0,1/2,0)	4,4	(1/2,1/2,0), (1/2,1/2,1)
**X_1^-**	(0,a)	*Ccce*	(0,0,1) (1,1,0) (-1,1,0)	(0,1/2,0)	2,4	(1/2,1/2,1)
**$^{\$}X_1^-$**	(a,b)	*Pban*	(1,1,0) (-1,1,0) (0,0,1)	(0,1/2,0)	4,8	(1/2,1/2,0), (1/2,1/2,1)
**X_2^+**	(a,a)	*P4/mbm*	(1,1,0) (-1,1,0) (0,0,1)	(1/2,1/2,0)	4,4	(1/2,1/2,0), (1/2,1/2,1)
**X_2^+**	(0,a)	*Cmce*	(0,0,1) (1,1,0) (-1,1,0)	(0,0,0)	2,4	(1/2,1/2,1)
**$^{\$}X_2^+$**	(a,b)	*Pbam*	(1,1,0) (-1,1,0) (0,0,1)	(0,0,0)	4,8	(1/2,1/2,0), (1/2,1/2,1)
**$^{\$\$}X_2^+/X_1^-$**	(0;a|b;0)	*Pcca*	(-1,1,0) (0,0,1) (1,1,0)	(1/4,1/4,1/4)	4,8	(1/2,1/2,0), (1/2,1/2,1)
**$^{\$\$}X_2^+/X_3^+$**	(0;a|b;0)	*Pbca*	(-1,1,0) (0,0,1) (1,1,0)	(0,0,0)	4,8	(1/2,1/2,0), (1/2,1/2,1)

$ These lower symmetry model were not considered.

$$ These models were used as a confirmatory check on the presence of only the X_2^+ mode at 685 °C (above T_C). For comparison these models produced χ^2 values of 6.17 and 6.30 (unstable); 76 and 75 variables, for *Pcca* and *Pbca* respectively. The best *P*4/*mbm* model gave χ^2 = 6.30 for 72 variables.

Space groups are given in the standard setting, which may result in switching of the axes compared to the *B*1*a*1 model.

OPD is the Order parameter direction. The parameters (s,i) represent, respectively, the *size* of the primitive unit cell of the subgroup relative to the parent space group and the *index* of the subgroup relative to the parent space group. For further details of these and other notations see the ISOTROPY website: stokes.byu.edu/iso/isodistorthelp.php

Table S2 Constraints applied to the 'simplified' *B*1*a*1 model discussed in the text, such that all atoms except O1/O1' confirm to the ideal *B*2*eb* model.

Atom 1	Parameter	Atom 2	Parameter
Bi1	x	Bi1a	x
Bi1	y	Bi1a	-y
Bi1	z	Bi1a	-z
Bi2	x	Bi2a	x
Bi2	y	Bi2a	-y
Bi2	z	Bi2a	-z
Ti2	x	Ti2a	x
Ti2	y	Ti2a	-y
Ti2	z	Ti2a	-z
O1	z	O1a	-z
O2	x	O2a	x
O2	y	O2a	-y
O2	z	O2a	-z
O3	x	O3a	x
O3	y	O3a	-y

O3	z	O3a	-z
O4	x	O4a	x
O4	y	O4a	-y
O4	z	O4a	-z
O5	x	O5a	x
O5	y	O5a	-y
O5	z	O5a	-z
O6	x	O6a	x
O6	y	O6a	-y
O6	z	O6a	-z

Note that the Ti2 y and z coordinates were also constrained to lie on the pseudo 2-fold axis.

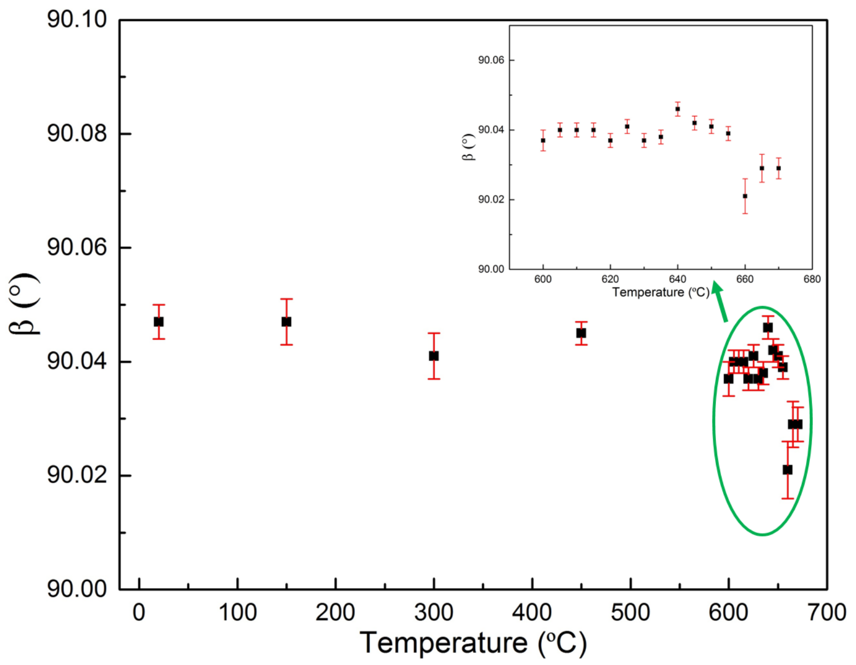

Figure S1: Temperature dependence of β angle for the monoclinic phase below T_C.

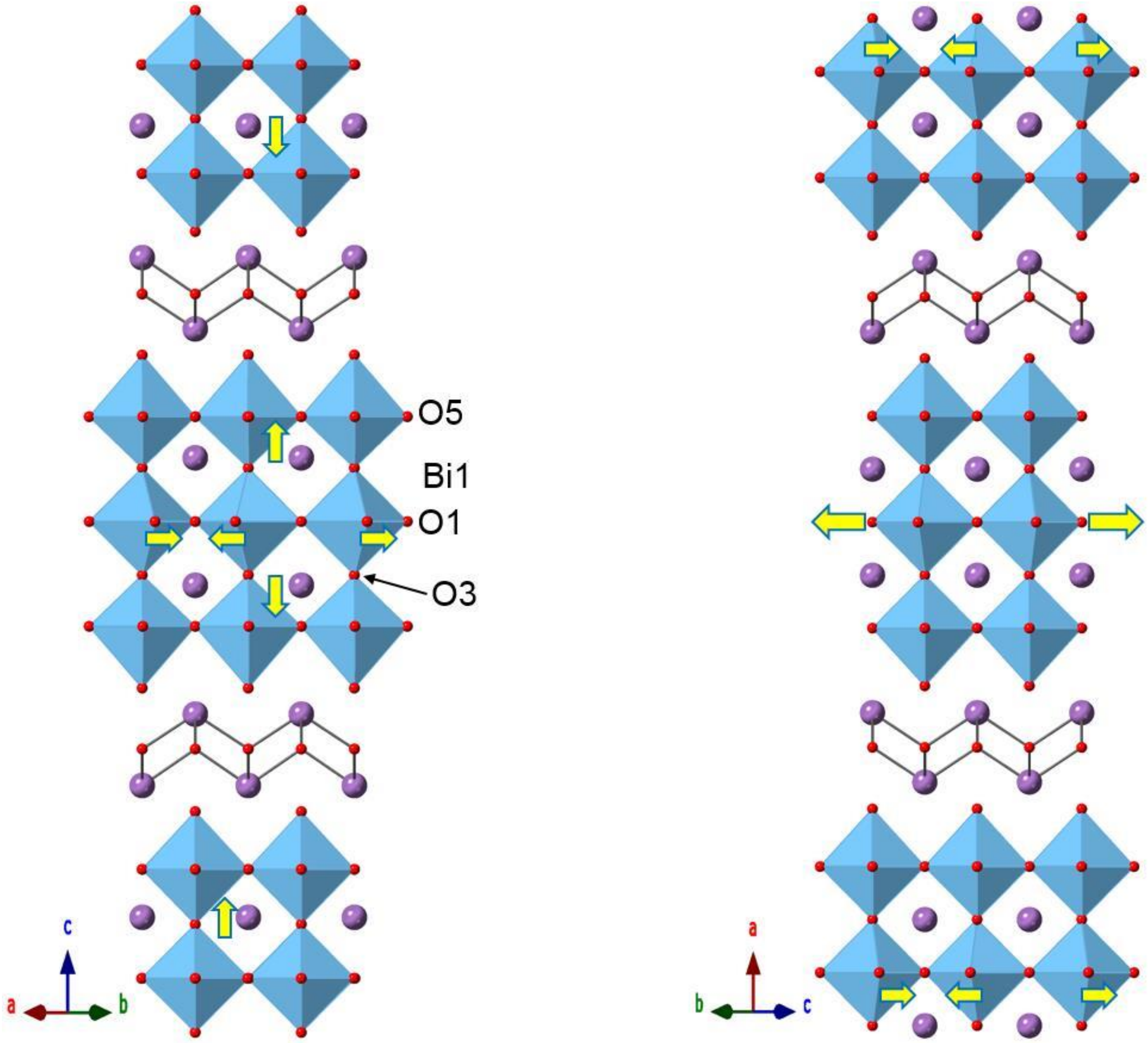

Figure S2: Crystal structures of the *P4/mbm* (left) and *Cmce* (right) models for the intermediate phase at 685 °C, with atomic numbering for the *P4/mbm* phase, and highlighting the differing X_2^+ modes and the M_1^+ modes for the *P4/mbm* model.

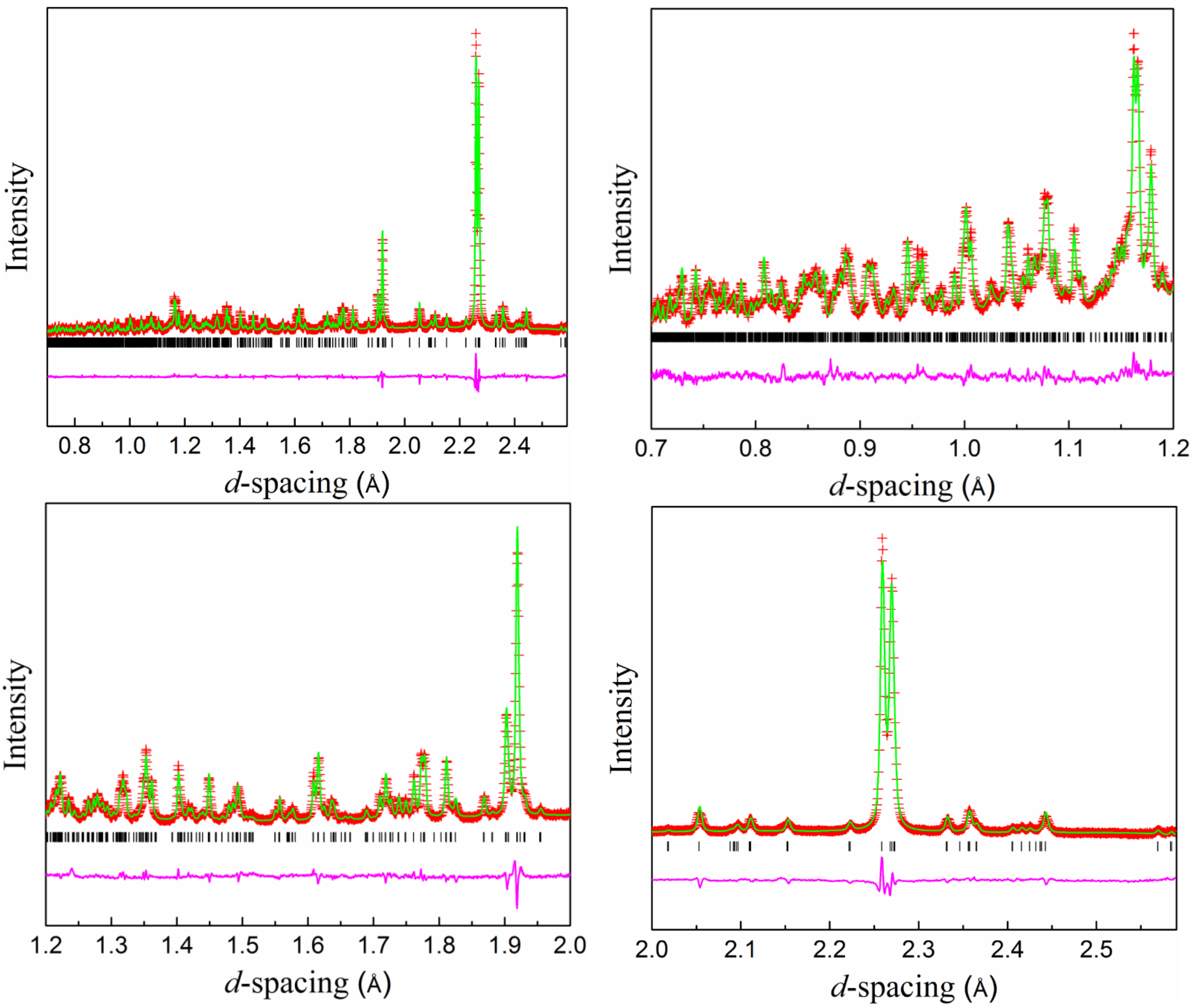

Figure S3. Full-range (top left) and expanded Rietveld plots (Bank 1), *B*1*a*1 ‘constrained’ model at 20 °C.

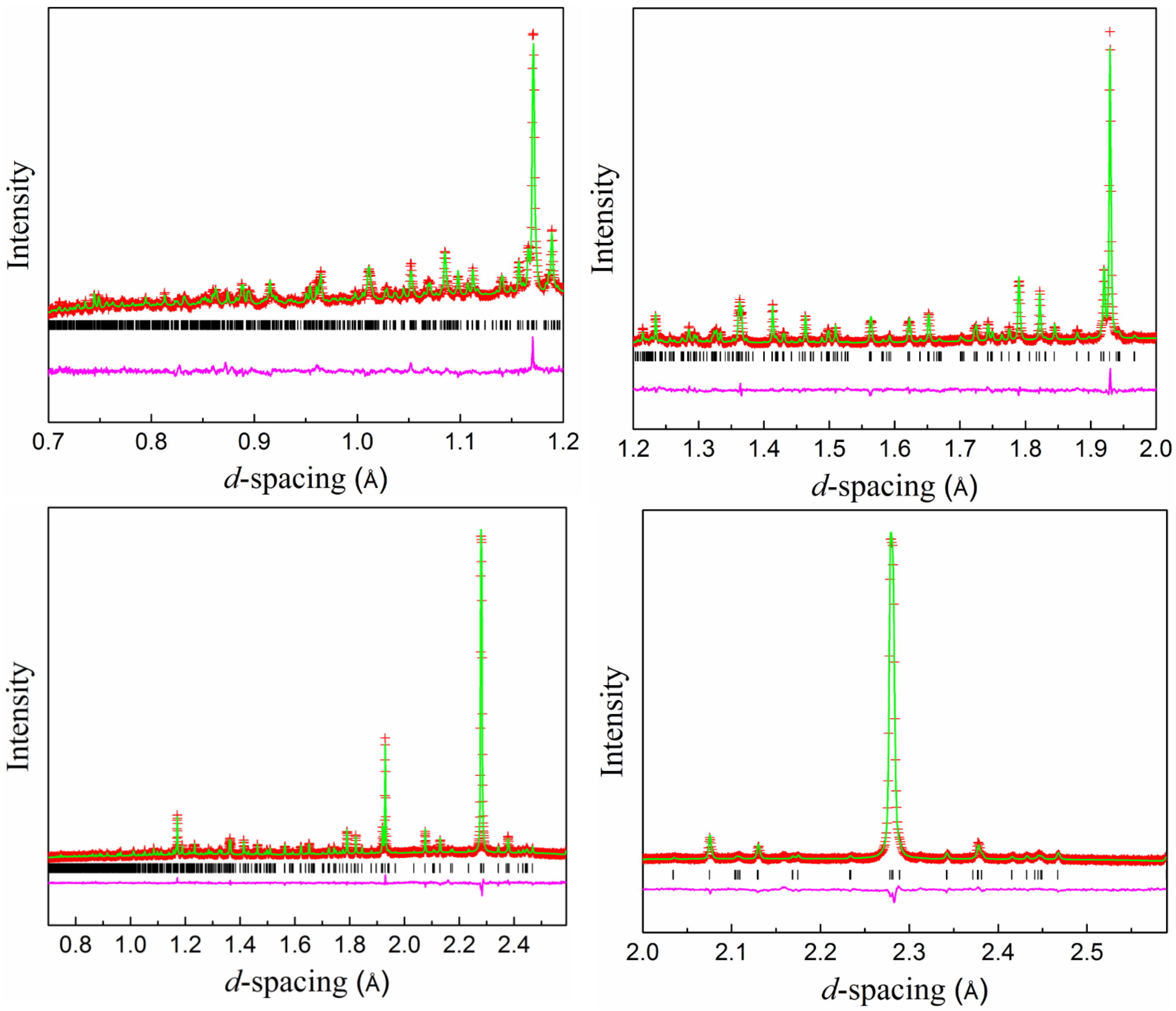

Figure S4. Full-range (top left) and expanded Rietveld plots (Bank 1), *B*1*a*1 'constrained' model at 655 °C.

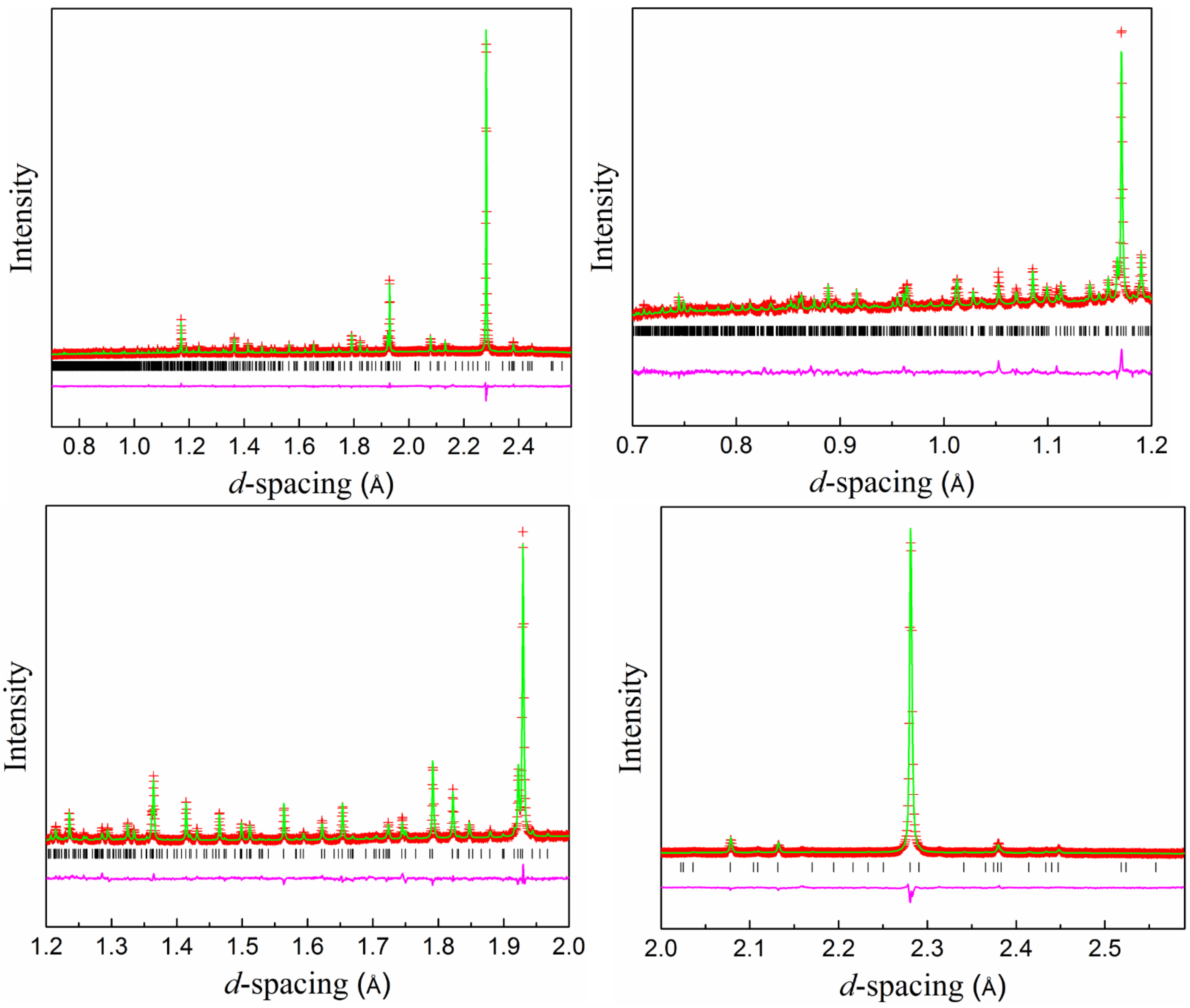

Figure S5. Full-range (top left) and expanded Rietveld plots (Bank 1), *P4/mbm* model at 685 °C.

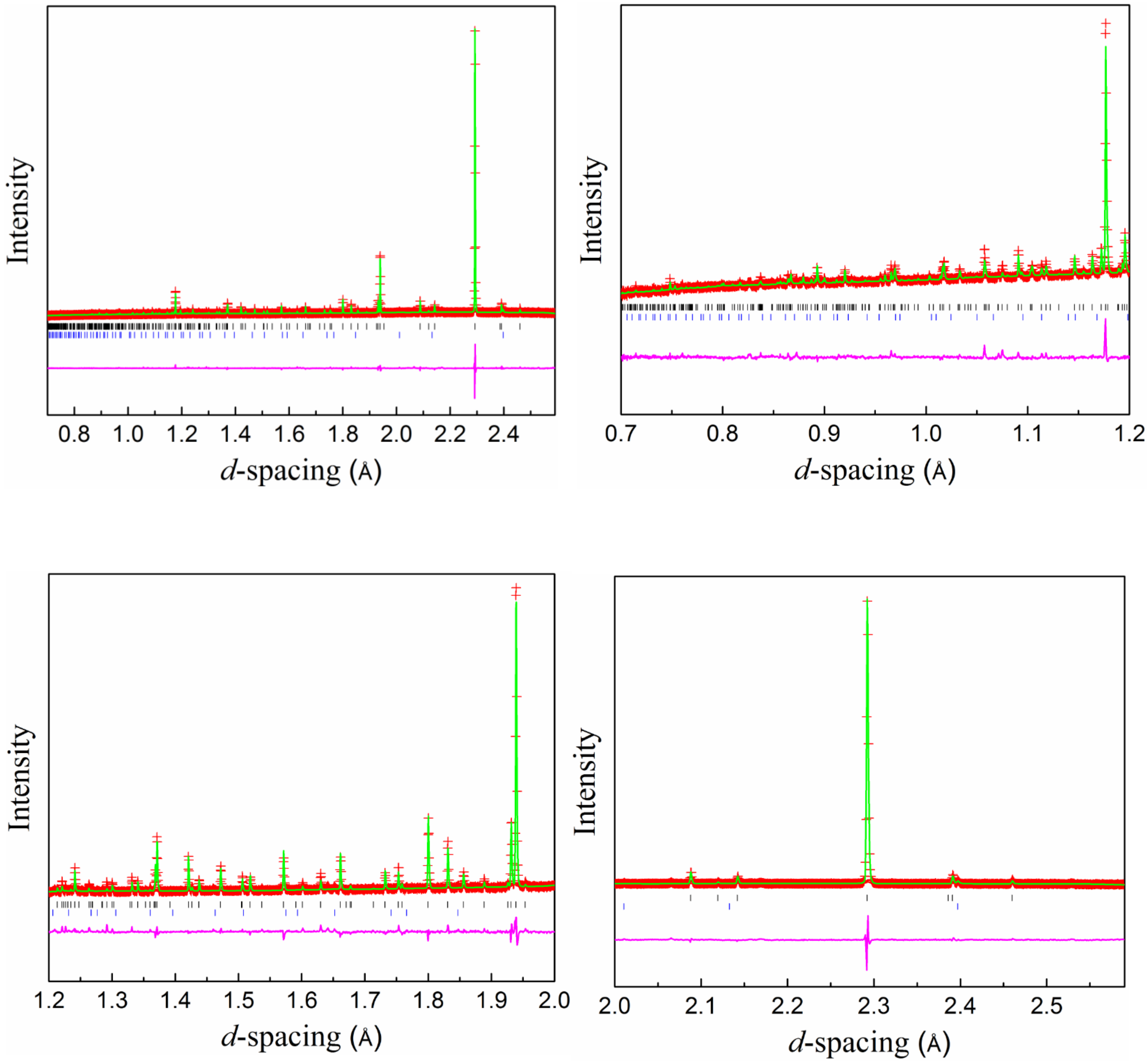

Figure S6. Full-range (top left) and expanded Rietveld plots (Bank 1), *I4/mmm* model (+ $Bi_2Ti_2O_7$ impurity) at 1000 °C.
